# Chemokine receptor expression and functional effects of chemokines on B cells: implication in the pathogenesis of rheumatoid arthritis

**DOI:** 10.1186/ar2823

**Published:** 2009-10-05

**Authors:** Toshihiro Nanki, Kazuki Takada, Yukiko Komano, Tomohiro Morio, Hirokazu Kanegane, Atsuo Nakajima, Peter E Lipsky, Nobuyuki Miyasaka

**Affiliations:** 1 Departments of Medicine and Rheumatology, Graduate School, Tokyo Medical and Dental University, 1-5-45, Yushima, Bunkyo-ku, Tokyo, 113-8519, Japan; 2 Department of Pharmacovigilance, Graduate School, Tokyo Medical and Dental University, 1-5-45, Yushima, Bunkyo-ku, Tokyo, 113-8519, Japan; 3 Department of Pediatrics and Developmental Biology, Graduate School, Tokyo Medical and Dental University, 1-5-45, Yushima, Bunkyo-ku, Tokyo, 113-8519, Japan; 4 Department of Pediatrics, Graduate School of Medicine, University of Toyama, 2630, Sugitani, Toyama, 930-0194, Japan; 5 Department of Joint Disease and Rheumatism, Nippon Medical School, 1-1-5, Sendagi, Bunkyo-ku, Tokyo, 113-8603, Japan; 6 Department of Rheumatology, Tokyo Metropolitan Police Hospital, 4-22-1, Nakano, Nakano-ku, Tokyo, 164-8541, Japan; 7 National Institute of Arthritis and Musculoskeletal and Skin Diseases, National Institutes of Health, 9000 Rockville Pike, Bethesda, MD 20892, USA; 8 Global Center of Excellence (GCOE) Program; International Research Center for Molecular Science in Tooth and Bone Diseases, Tokyo Medical and Dental University, 1-5-45, Yushima, Bunkyo-ku, Tokyo, 113-8519, Japan

## Abstract

**Introduction:**

Accumulation of B cells in the rheumatoid arthritis (RA) synovium has been reported, and it has been thought that these cells might contribute to the pathogenesis of RA by antigen presentation, autoantibody production, and/or inflammatory cytokine production. Chemokines could enhance the accumulation of B cells in the synovium. The aims of this study were to determine chemokine receptor expression by B cells both in the peripheral blood of normal donors and subjects with RA, and at the inflammatory site in RA, and the effects of chemokines on B cell activation.

**Methods:**

Cell surface molecule expression was analyzed by flow cytometry. Cellular migration was assessed using chemotaxis chambers. Cellular proliferation was examined by ^3^H-thymidine incorporation. Tumor necrosis factor (TNF) production was assayed by enzyme-linked immunosorbent assay.

**Results:**

Significant numbers of peripheral blood B cells of healthy donors and subjects with RA expressed CC chemokine receptor (CCR)5 and CXCR3, and most B cells expressed CCR6, CCR7, CXCR4 and CXCR5. CCR5 expression was more frequent on CD27^+ ^than CD27^- ^peripheral blood B cells of healthy donors and RA. Synovial B cells more frequently expressed CCR5, but less often expressed CCR6, CCR7 and CXCR5 compared to peripheral blood in RA. Further functional analyses were performed on peripheral blood B cells from healthy donors. Migration of peripheral blood B cells, especially CD27^+ ^B cells, was enhanced by CC chemokine ligand (CCL)20, CCL19, CCL21 and CXCL12. All four chemokines alone induced B cell proliferation; with CCL21 being the most effective. CCL21 also enhanced the proliferation of anti-immunoglobulin (Ig)M-stimulated B cells and blockade of CCR7 inhibited this effect. CCL20, CCL21 and CXCL12 enhanced TNF production by anti-IgM mAb-stimulated B cells. Finally, stimulation with CXCL12, but not CCL20, CCL19 and CCL21, enhanced inducible costimulator-ligand (ICOSL) expression by peripheral blood B cells of healthy donors and RA, but did not increase B cell-activating factor receptor or transmembrane activator and CAML-interactor.

**Conclusions:**

The data suggest that CCR5, CCR6, CCR7, CXCR3, CXCR4 and CXCR5 may be important for the B cell migration into the synovium of RA patients, and also their local proliferation, cytokine production and ICOSL expression in the synovium.

## Introduction

Rheumatoid arthritis (RA) is characterized by chronic inflammation of multiple joints. As B cell depletion by treatment with rituximab, an anti-CD20 monoclonal antibody (mAb), is beneficial for RA patients [1,2], B cells are considered to play important roles in the pathogenesis of RA. In this regard, the synovial tissue of RA patients shows abundant accumulation of inflammatory cells, including T cells, macrophages, dendritic cells and B cells [3-6]. Synovial B cells could present antigens to T cells. Importantly, rheumatoid factor-expressing B cells that are found within the synovium [7] can present any antigen in the context of an immune complex and, thereby, trigger T cells specific for a variety of foreign antigens [8]. Notably, the severity of RA correlates with levels of rheumatoid factor [9]. Furthermore, activated B cells produce inflammatory cytokines, such as TNF [10]. Therefore, synovial B cells could contribute to the pathogenesis of RA by antigen presentation, autoantibody production, and inflammatory cytokine production. One of the mechanisms for accumulation of B cells in synovial tissues relates to the interaction with chemokines produced in the RA synovium and chemokine receptors expressed by the B cells [6].

Chemokines are classified into C, CC, CXC, and CX3C subclasses based on the conserved cysteine motifs [11], and are involved in cellular migration, activation of adhesion molecules, cellular proliferation, cytokine production and regulation of apoptosis [12,13]. Chemokines contribute to homeostatic migration as well as entry into acute and chronic inflammatory sites. Expression of chemokines and chemokine receptors in the RA synovial tissue has been extensively analyzed, and chemokines are thought to be potential therapeutic targets [14,15]. However, the role of chemokines specifically on B cells in RA has not been completely delineated.

In this study, we examined chemokine receptor expression by peripheral blood in both normal donors and subjects with RA, and also synovial B cells from subjects with RA, and determined the functional effects of chemokines on B cells.

## Materials and methods

### Samples

Peripheral blood samples were obtained from healthy donors and subjects with RA after obtaining informed consent. RA was diagnosed according to the criteria of the American College of Rheumatology [16]. Synovial tissues were obtained at the time of total knee joint replacement from RA patients. Signed consent forms were obtained prior to the operation. The study protocol was approved in advance by the Ethics Committee of the Tokyo Medical and Dental University.

### Chemokine receptor expression

Peripheral blood mononuclear cells (PBMCs) were isolated by ficoll-hypaque (Immuno-Biological Laboratories, Gunma, Japan) gradient centrifugation. The synovial tissue was minced and incubated with 0.3 mg/ml collagenase (Sigma, St. Louis, MO, USA) for one hour at 37°C in Dulbecco's Modified Eagle Medium (DMEM) (Sigma, St. Louis, MO, USA). Partially digested pieces of the tissue were pressed through a metal screen to obtain single cell suspensions. The following mAbs were used for FACS analysis: phycoerythrin (PE) Cy5-conjugated anti-CD19 mAb (J4.119; Beckman Coulter, San Jose, CA, USA), fluorescein isothiocyanate (FITC)-conjugated anti-CD27 (M-T271; Ancell, Bayport, MN, USA) mAb, PE-conjugated anti-CC chemokine receptor (CCR)5 (2D7; BD Bioscience, San Jose, CA, USA), -CCR6 (53103; R&D Systems, Minneapolis, MN, USA), -CCR7 (150503; R&D Systems, Minneapolis, MN, USA), -CXCR3 (49801; R&D Systems, Minneapolis, MN, USA), -CXCR4 (12G5; R&D Systems, Minneapolis, MN, USA) and -CXCR5 (51505.111; R&D Systems, Minneapolis, MN, USA) mAbs, and isotype-matched control mAbs. PBMCs or synovial tissue cells were incubated with the mAbs for 20 minutes, and then rinsed with PBS-3% fetal calf serum (FCS; Sigma, St. Louis, MO, USA). More than 5000 stained cells were analyzed with a FACSCalibur (BD Bioscience, San Jose, CA, USA).

### Migration assay

Cell migration was assessed in 24-well chemotaxis chambers (6.5 mm diameter, 5 μm pore polycarbonate transwell culture insert; Costar, Cambridge, MA, USA). ECV304 cells (2 × 10^5^) were cultured in the chemotaxis chambers for 48 to 72 hours in medium 199 (Sigma, St. Louis, MO, USA) with 10% FCS. The migration medium (Roswell Park Memorial Institute (RPMI)1640 medium (Sigma, St. Louis, MO, USA):medium 199 = 1:1, 0.5% BSA) supplemented where indicated with various concentrations of chemokines (CC chemokine ligand (CCL)20, CCL19, CCL21, and CXCL12: PeproTech, Rocky Hill, NJ, USA) was added to the lower wells. ECV304 coated chemotaxis chambers were placed in each well, and 5 × 10^5 ^PBMCs suspended in migration medium were added to the upper wells. After two hours of incubation, the membrane was removed, and migrated cells were stained with PE Cy5-conjugated anti-CD19 mAb (J4.119) and FITC-conjugated anti-CD27 mAb (M-T271). The cells were counted by FACSCalibur.

### Proliferation assay

Peripheral blood CD19^+ ^B cells were purified by magnetic-activated cell sorting microbead-coupled mAb and magnetic cell separation columns (Miltenyi Biotec, Auburn, CA, USA). Purity of CD19^+ ^B cells was determined by flow cytometry, and was more than 95%. To block CCR7, B cells were incubated with 5 μg/ml anti-CCR7 mAb (150503; R&D Systems, Minneapolis, MN, USA) or control mAb for 30 minutes. Then, the 5 × 10^5 ^B cells were incubated in 96-well with the indicated chemokines with or without pre-coated anti-IgM mAb (2 μg UHB; SouthernBiotech, Birmingham, AL, USA) in RPMI1640 with 10% FCS at 37°C for 48 hours. ^3^H-thymidine (1 μCi; Amersham Biosciences, Little Chalfont, Buckinghamshire, UK) was added and the B cells were incubated for 24 hours. Afterward, the incorporated radioactivity was quantified. After the 72-hour incubation, viabilities of the cells, determined by trypan blue exclusion, were 87.3% and 80.3% without and with anti-IgM stimulation, respectively.

### TNF production

Purified 5 × 10^5 ^peripheral blood B cells were stimulated with the indicated chemokines with or without coating of wells with anti-IgM mAb (2 μg UHB) in 96-well in RPMI1640 with 10% FCS at 37°C for 24 hours. Afterward, the concentration of TNF in the culture supernatant was assayed using an ultra sensitive ELISA kit (BioSource International, Camarillo, CA).

### Cell surface molecule expression

PBMCs were cultured with the indicated chemokine in RPMI1640+10% FCS for 24 hrs. Afterward, the cells were stained with PE Cy5-conjugated anti-CD19 mAb (J4.119) and FITC-conjugated anti-inducible costimulator-ligand (ICOSL) mAb (MIH12; eBioscience, San Diego, CA, USA), PE-conjugated anti-B cell-activating factor receptor (BAFF-R; 8A7; eBioscience, San Diego, CA, USA), -transmembrane activator and CAML-interactor (TACI) mAb (11H3; eBioscience, San Diego, CA, USA), or isotype-matched control mAb. The stained cells were analyzed with a FACSCaliber.

### Statistical analysis

Paired *t *test was used to compare paired samples of CD27^- ^and CD27^+ ^peripheral blood B cells, and peripheral blood and synovial B cells from the same subjects for chemokine receptor expression and migration. Differences in migration, fold increase of proliferation and TNF production were examined for statistical significance using the unpaired *t *test. All data were expressed as mean ± standard error of the mean (SEM). A *P *value less than 0.05 denoted the presence of a statistically significant difference.

## Results

### Chemokine receptor expression by B cells

**Figure 1:**
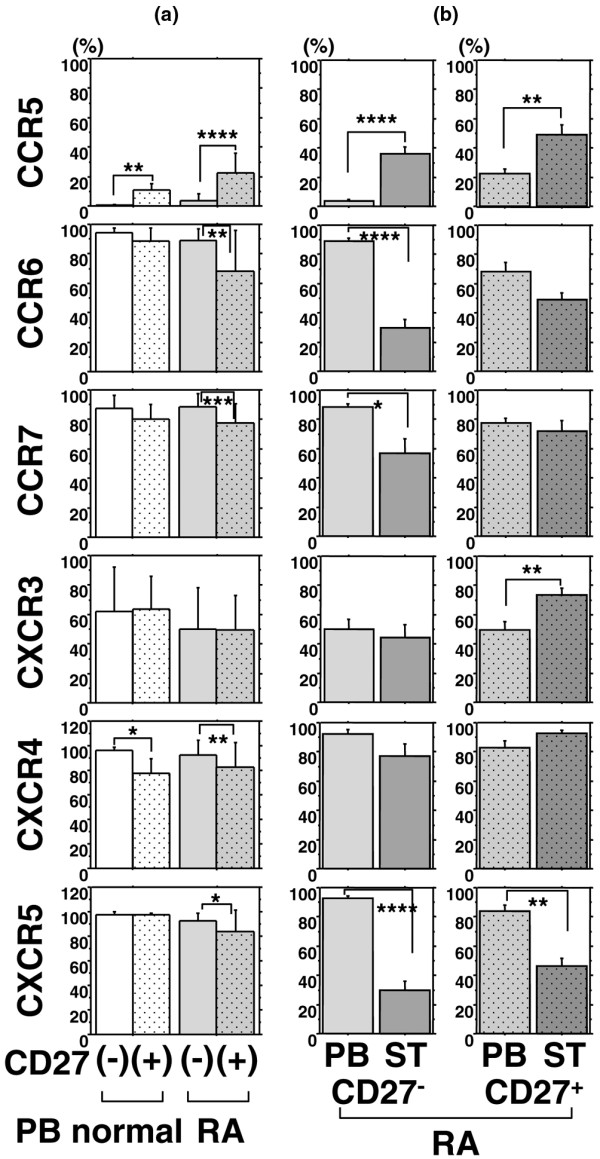
Chemokine receptor expression by B cells. Peripheral blood mononuclear cells (PBMCs) from healthy donors (n = 4 to 7) and rheumatoid arthritis (RA) patients (n = 17 to 18) and synovial cells from RA patients (n = 10 to 11) were stained with CD19, CD27, and CCR5, CCR6, CCR7, CXCR3, CXCR4 or CXCR5, and the expression of the various markers was analyzed by flow cytometry. CD19^+ ^B cells were gated, and the frequency of expression of each chemokine receptor is shown. Data represent mean ± standard error of the mean. *P < 0.05, **P < 0.01, ***P < 0.001, ****P < 0.0001. ST = synovial tissue.

Chemokine receptor expression by naive CD27^- ^B cells and memory CD27^+ ^B cells from the peripheral blood of healthy donors was analyzed by flow cytometry. As shown in Figure 1a, most peripheral blood B cells of healthy donors expressed CCR6, CCR7, CXCR4 and CXCR5. About 60% of the B cells expressed CXCR3, and less than 20% of the B cells expressed CCR5. These results are similar to previous reports [17-20]. We compared the chemokine receptor expression between CD27^- ^and CD27^+ ^B cells. The frequencies of CCR6, CCR7, CXCR3 and CXCR5 expression were not different between CD27^- ^and CD27^+ ^B cells of normal donors. However, the proportion of CCR5-expressing peripheral blood CD27^+ ^B cells was significantly higher than that of CD27^- ^B cells in normal controls. The percentage of CD27^+ ^B cells expressing CXCR4 was less than CXCR4-expressing CD27^- ^B cells in normal controls.

Next, we analyzed the chemokine receptor expression by CD27^- ^B cells and CD27^+ ^B cells from peripheral blood and synovial tissue of subjects with RA. The frequency of CD27-expressing peripheral blood B cells was not significantly different between subjects with RA and healthy donors (data not shown). The proportion of the chemokine receptor expression of RA peripheral blood B cells was similar to that of healthy donors without any statistically significant differences. As with healthy donors, CCR5 expression by RA peripheral blood CD27^+ ^B cells was more frequent than that of CD27^- ^B cells, and CXCR4 expression by CD27^+ ^B cells was less frequent than that of CD27^- ^B cells. In addition, the proportions of CCR6, CCR7 and CXCR5 expression were significantly less by CD27^+ ^compared with CD27^- ^B cells in subjects with RA.

**Figure 2:**
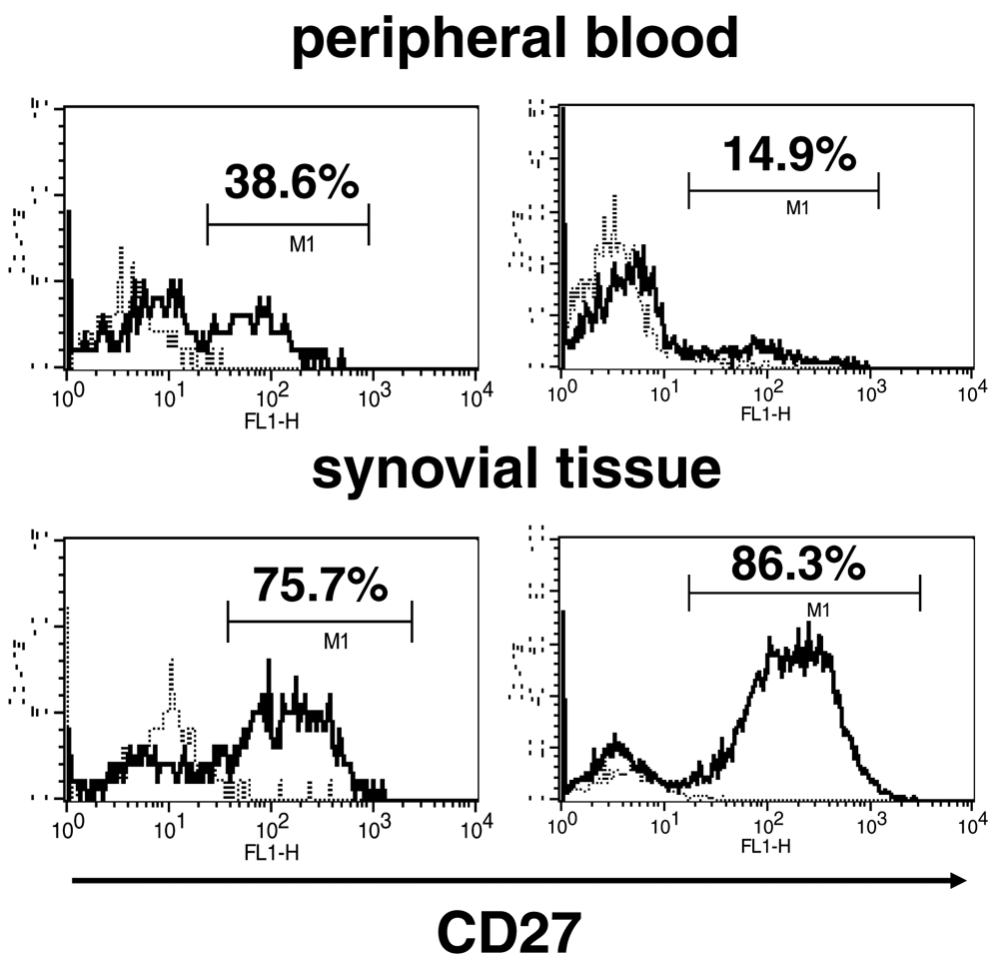
CD27 expression by peripheral blood and synovial tissue B cells of subjects with RA. CD19^+ ^B cells were gated, and representative histograms from two patients with rheumatoid arthritis (RA) show the cells stained with anti-CD27 monoclonal antibody (mAb) (solid lines) and isotype-matched control (dotted lines).

We also compared the chemokine receptor expression between peripheral blood and synovial tissue B cells of RA (Figure 1b). The frequency of CD27^+ ^by synovial B cells was significantly higher than that of peripheral blood B cells in RA subjects (Figure 2) (peripheral blood, 30.0 ± 5.1% (mean ± SEM); synovial B cells, 62.3 ± 4.7%; *P *< 0.005, n = 11), as we have previously reported [21], suggesting that a specific subset of B cells might be recruited to the inflammatory site in RA. The proportion of synovial B cells that expressed CCR5 was significantly higher than that of either peripheral blood CD27^- ^or CD27^+ ^B cells of subjects with RA. The proportion of CXCR3-expressing CD27^+ ^B cells in the synovium was higher than peripheral blood. In addition, the frequency of synovial B cells that expressed CCR6 and CCR7 was less than that expressed by peripheral blood CD27^- ^B cells, but not CD27^+ ^B cells. The proportion of synovial B cells that expressed CXCR5 was less than that in peripheral blood. CXCR4 expression was no different between peripheral blood and synovial B cells.

### Migration

**Figure 3:**
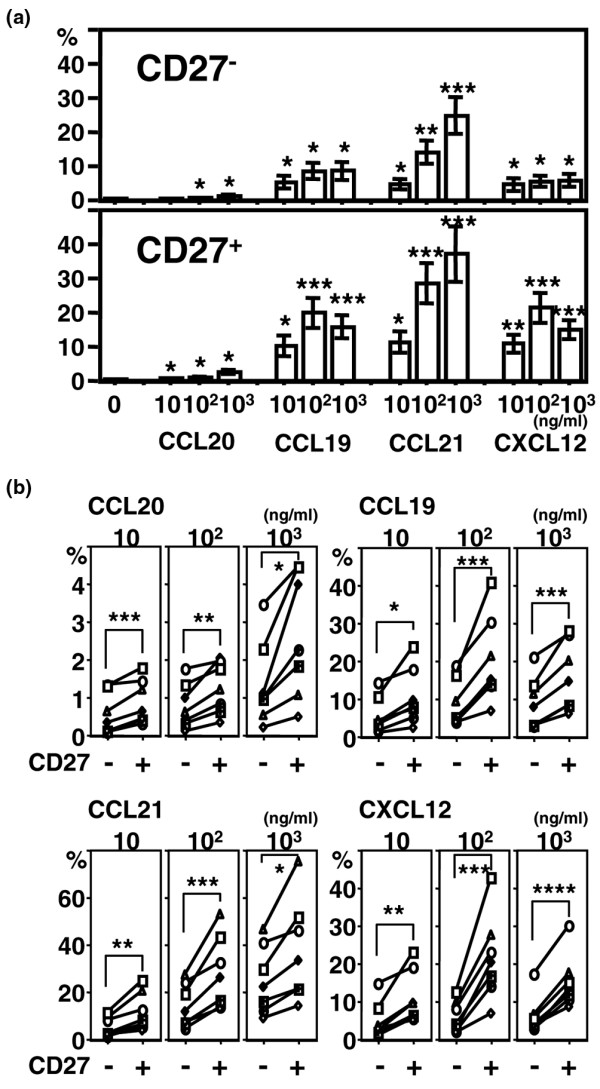
B cell migration in response to chemokines. Peripheral blood mononuclear cells (PMBCs) from healthy donors were cultured in the presence of various concentrations of CCL20, CCL19, CCL21, or CXCL12 for two hours. The cells migrated through ECV304-coated transwells were stained with CD19 and CD27, and the numbers of cells were assessed. The percentage of migrated cells was calculated by dividing the number of migrated CD27^- ^or CD27^+ ^B cells by the number of total cultured CD27^- ^or CD27^+ ^B cells for six to seven donors. **(a) **Values are mean ± standard error of the mean. *P < 0.05, **P < 0.01, ***P < 0.005, vs no chemokine. **(b) **Each symbol represents an individual subject. *P < 0.05, **P < 0.01, ***P < 0.005, ****P < 0.0001.

As frequencies of the analyzed chemokine receptor expression by peripheral blood B cells were not significantly altered by RA, we next examined functional effects of chemokine ligands for the chemokine receptors using peripheral blood B cells of healthy donors. Most peripheral B cells expressed CCR6, CCR7 and CXCR4, and a significant number of RA synovial B cells expressed also them. Therefore, we selected four chemokines, CCL20, a ligand for CCR6, CCL19 and CCL21, ligands for CCR7, and CXCL12, a ligand for CXCR4. First, we analyzed the effects the chemokines on migration of peripheral blood B cells. Each of the four chemokines induced migration of both CD27^- ^and CD27^+ ^B cells (Figure 3a). However, the migration induced by CCL21 was most prominent. Comparison of the migratory effects of the chemokines on peripheral blood CD27^- ^and CD27^+ ^B cells in each individual showed that for each of the chemokines, the chemotactic response of CD27^+ ^B cells was significantly greater than with CD27^- ^B cells (Figure 3b).

### Proliferation

**Figure 4:**
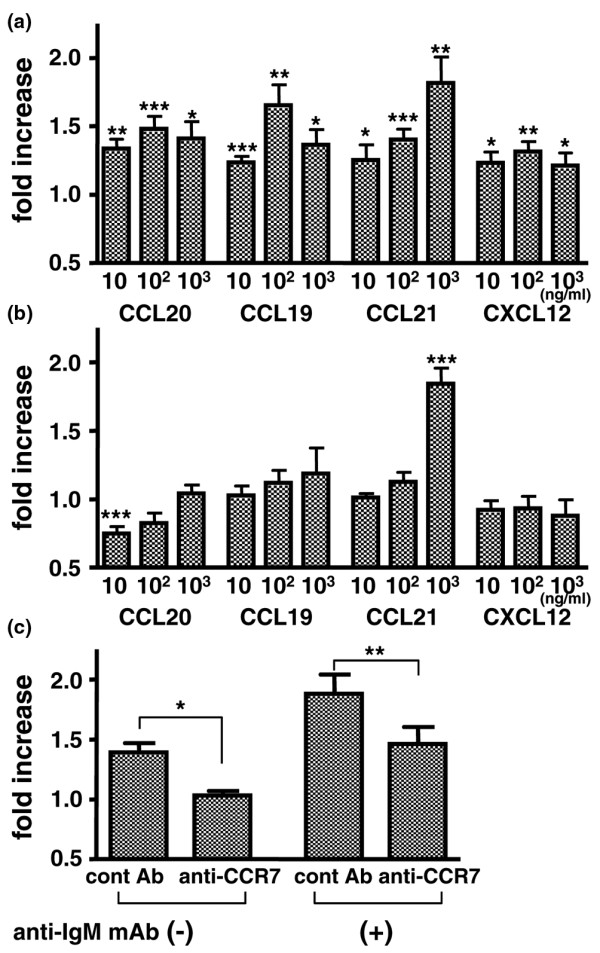
B cell proliferation in response to chemokine stimulation. Purified B cells from peripheral blood mononuclear cells (PBMCs) of normal donors were stimulated with the indicated chemokines for 48 hours **(a) **without and **(b) **with anti-IgM stimulation. **(c) **To block CCR7, the B cells were pre-incubated with anti-CCR7 monoclonal antibody (mAb) or control mAb for 30 minutes. ^3^H-thymidine was added and B cells were incubated for 24 hours. The incorporated radioactivity was quantified. Fold increase in ^3^H-thymidine incorporation in response to chemokine stimulation for four to eight donors was calculated. Values are mean ± standard error of the mean. (a, b) *P < 0.05, **P < 0.005, ***P < 0.0005, vs no chemokine stimulation. (c) *P < 0.05, **P < 0.005.

The effect of chemokines on B cell proliferation was next analyzed in normal donors. Although the effect was weak, CCL20, CCL19, CCL21 and CXCL12 induced significant B cell proliferation (Figure 4a). Among the chemokines, 1000 ng/ml CCL21 was the most effective stimulus of proliferation. Stimulation with anti-IgM mAb induced B cell proliferation (fold increase: 5.5 ± 0.8). Stimulation with a low concentration of CCL20 (10 ng/ml) decreased the proliferation of anti-IgM-stimulated peripheral blood B cells. In contrast, a high concentration of CCL21 (1000 ng/ml) significantly enhanced the anti-IgM-stimulated B cell proliferation (Figure 4b). Notably, CCL21-induced proliferation was inhibited by anti-CCR7 mAb by blocking the corresponding receptor (Figure 4c).

### TNF production

**Figure 5:**
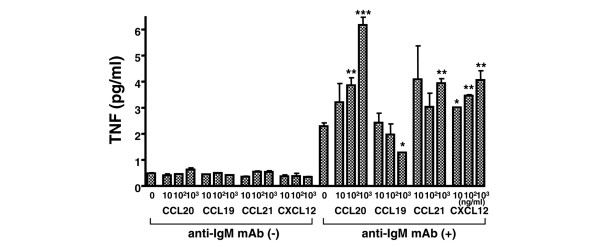
TNF production by chemokine stimulation. Purified peripheral blood B cells from normal donors were incubated with the indicated chemokines with or without anti-IgM monoclonal antibody (mAb) for 24 hours. The concentration of TNF in the culture supernatant was measured by ELISA. Data are mean ± standard error of the mean values of three independent experiments analyzed in duplicate. *P < 0.05, **P < 0.005, ***P < 0.0005, vs no chemokine stimulation.

We also analyzed the effect of chemokine stimulation on TNF production by peripheral blood B cells of healthy donors. Without anti-IgM stimulation, B cells secreted small amounts of TNF (less than 1 pg/ml by this assay), and stimulation with CCL20, CCL19, CCL21 and CXCL12 did not change the TNF production (Figure 5). In contrast, anti-IgM mAb stimulation increased TNF production by B cells. Moreover, co-stimulation of anti-IgM activated B cells by CCL20, CCL21, and CXCL12 enhanced TNF production, whereas CCL19 decreased TNF production.

### Cell surface molecule expression

**Figure 6:**
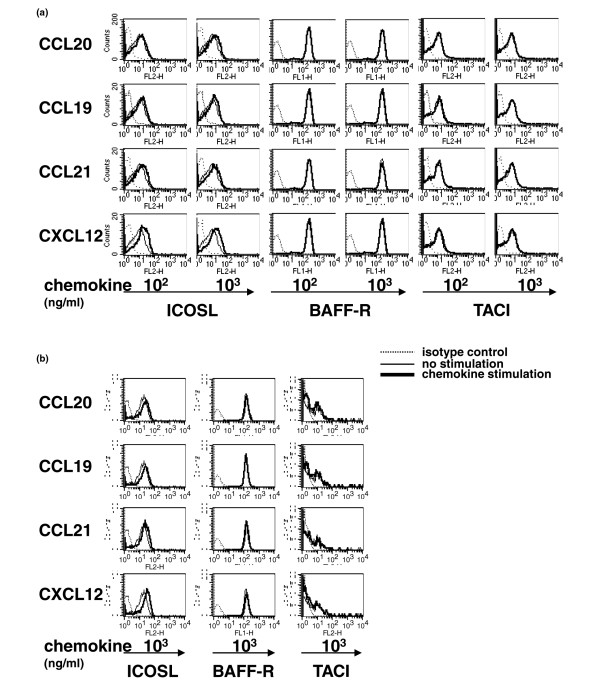
Cell surface expression of ICOSL and BAFF receptors. Peripheral blood mononuclear cells (PBMCs) from **(a) **healthy donors and **(b) **subjects with rheumatoid arthritis (RA) were stimulated with the indicated chemokines for 24 hours. Afterward, the cells were stained with monoclonal antibody (mAbs) to CD19 and inducible costimulator-ligand (ICOSL), B cell-activating factor receptor (BAFF-R) or transmembrane activator and CAML-interactor (TACI), and the expression was analyzed by flow cytometry. Representative expression patterns by CD19^+ ^cells are shown from three similar independent experiments.

Finally, we examined the effects of the chemokines on the expression of the cell surface molecules ICOSL, BAFF-R and TACI by peripheral blood B cells of normal donors and subjects with RA. ICOSL was expressed by unstimulated peripheral B cells of both normals and subjects with RA, and CXCL12 enhanced the expression of ICOSL on both normal and RA B cells. In contrast, the effect of CCL20, CCL19 and CCL21 was not significant (Figures 6a and 6b). BAFF-R and TACI were also expressed by unstimulated peripheral B cells of normal donors and subjects with RA. However, stimulation with either CCL20, CCL19, CCL21 or CXCL12 did not alter expression.

## Discussion

In this study, we showed that significant numbers of peripheral blood and RA synovial B cells express CCR5, CCR6, CCR7, CXCR3, CXCR4, and CXCR5. The ligand chemokines, CCL3, CCL4 and CCL5 for CCR5, CCL20 for CCR6, CCL19 and CCL21 for CCR7, CXCL9, CXCL10 and CXCL11 for CXCR3, CXCL12 for CXCR4, and CXCL13 for CXCR5 has been reported to be expressed in the RA synovium [22-29]. Therefore, interactions between the chemokines and the chemokine receptors might contribute to B cell migration into the synovial tissue in patients with RA.

In the RA synovium, the proportion of memory CD27^+ ^B cells was increased compared with peripheral blood of RA patients. The results also showed that CCR5 was expressed more frequently by peripheral blood CD27^+ ^B cells compared with CD27^-^, and the proportion of synovial B cells expressing CCR5 was increased compared with peripheral blood. These results suggest that interaction between CCR5 and the ligand chemokines could contribute to the accumulation of CD27^+ ^B cells in the synovium. Alternatively, because the migration of CD27^+ ^B cells to all the chemokines analyzed was greater than that of CD27^- ^B cells, the increased proportion of CD27^+ ^B cells in the synovium might be related to their higher chemotactic activity. In contrast, the expression of CCR6, CCR7 and CXCR5 was downregulated by the synovial B cells. As most peripheral blood B cells express these chemokine receptors, it is not likely that the chemokine receptor-negative B cells selectively migrated into the synovium. Rather chemokine receptor expression might be downregulated after ligation of the corresponding ligand chemokine. Alternatively, stimulation with cytokines or adhesion molecules may downregulate chemokine receptor expression in the synovium.

The present study showed that stimulation with chemokine regulates peripheral blood B cell proliferation. Previous studies showed the presence of germinal center-like structures in the RA synovium [30], somatic hypermutation of the Ig variable region genes, B cell clonal expansion, and a skewed Ig repertoire in the synovium [31,32]. Collectively, these results suggest that synovial B cells might be antigenically stimulated at the inflammatory site. Based on such B cell stimulation in the synovium, the interaction between chemokines and chemokine receptors, especially CCL21 and CCR7, might also contribute to B cell proliferation. There is an evidence that follicular dendritic cells in the RA synovium produce CXCL13, a ligand for CXCR5 [29]. Interaction with the expressed CXCL13 and CXCR5 on B cells might contribute to the formation of the germinal center-like structures in the synovium.

Stimulation with CCL20, CCL21 and CXCL12 enhanced TNF production by anti-IgM mAb-stimulated peripheral blood B cells suggesting that chemokine stimulation in the RA synovium might also increase TNF production by synovial B cells. It is widely known that TNF plays important roles in the pathogenesis of RA and blockade of this cytokine is an effective therapy for RA [33]. Moreover, CXCL12 upregulated ICOSL expression on peripheral blood B cells. ICOSL could interact with inducible costimulator (ICOS), which is expressed by activated T cells [34]. We showed previously that ICOS expression was upregulated on RA synovial T cells [35]. Thus, upregulated ICOSL on CXCL12-stimulated B cells could augment T cell stimulation in the synovium. Taken together, interaction between chemokine and chemokine receptor might play roles not only on B cell migration into the synovium, but also B cell activation in the synovium. In this regard, we reported previously that CXCL12 enhanced cellular proliferation and expression of cytokines and activation markers by peripheral blood T cells [36,37], and that CCL2, CCL5 and CXCL12 upregulated the expression of cytokines and chemokines by fibroblast-like synoviocytes from RA [38]. Thus, chemokine stimulation in the RA synovial tissue could play an important role on the chronic immune activation found in this tissue.

## Conclusions

CCR5, CCR6, CCR7, CXCR3, CXCR4, and CXCR5 might be important for B cell migration into the synovium of RA. Chemokines are suggested to contribute to B cell migration as well as their proliferation, cytokine production and ICOSL expression in the RA synovium.

## Abbreviations

BAFF-R: B cell-activating factor receptor; BSA: bovine serum albumin; CCL: CC chemokine ligand; CCR: CC chemokine receptor; DMEM: Dulbecco's Modified Eagle Medium; ELISA: enzyme-linked immunosorbent assay; FCS: fetal calf serum; FITC: fluorescein isothiocyanate; ICOS: inducible costimulator; ICOSL: inducible costimulator-ligand; Ig: immunoglobulin; mAb: monoclonal antibody; PBMCs: peripheral blood mononuclear cells; PBS: phosphate-buffered saline; PE: phycoerythrin; RA: rheumatoid arthritis; RPMI: Roswell Park Memorial Institute; SEM: standard error of the mean; TACI: transmembrane activator and CAML-interactor; TNF: tumor necrosis factor.

## Competing interests

The authors declare that they have no competing interests.

## Authors' contributions

TN designed the study, and carried out data analysis, interpretation, and manuscript preparation. KT and YK participated in the data analysis and interpretation, and assisted in manuscript preparation. TM, HK, AN, PEL, and NM assisted in data interpretation and manuscript preparation. All authors read and approved the final manuscript.
